# Supplementary material for: A Novel Missense Variant Associated with A Splicing Defect in A Myopathic Form of PGK1 Deficiency in The Spanish Population
Source: Genes (Basel). 2019 Oct 10;10(10):785. doi: 10.3390/genes10100785 (PMC6826351; doi:10.3390/genes10100785)
Supplement: Supplementary file 1 [file genes-10-00785-s001.pdf]

Table S1: Next-generation sequencing (NGS) panel of 32 genes associated with inherited metabolic myopathies.

Gene	Reference	Coverage (%)
AGL	NM_000642	100
ALDOA	NM_001243177	98.26
ABHD5	NM_016006	100
ACADL	NM_001608	100
AMPD1	NM_000036	100
ENO3	NM_053013	96.52
GBE1	NM_000158	100
ACADM	NM_001127328	100
ACADS	NM_000017	99.51
ATP2A1	NM_173201	95.62
GYG1	NM_004130	100
GYS1	NM_002103	100
ACADVL	NM_000018	94.41
CPT1B	NM_004377	95.89
LDHA	NM_001165414	99.63
PFKM	NM_001166686	100
CPT2	NM_000098	100
ETFA	NM_000126	100
PGAM2	NM_000290	100
ETFB	NM_001014763	100
ETFDH	NM_004453	100
PGK1	NM_000291	100
HADHA	NM_000182	100
PGM1	NM_001172818	100
HADHB	NM_000183	98.83
PHKA1	NM_002637	100
LPIN1	NM_001261428	100
SLC22A5	NM_003060	97.97
PHKB	NM_000293	100
PNPLA2	NM_020376	100
SLC25A20	NM_000387	100
PYGM	NM_005609	100
